# Serotonin Decreases the Gain of Visual Responses in Awake Macaque V1

**DOI:** 10.1523/JNEUROSCI.1339-17.2017

**Published:** 2017-11-22

**Authors:** Lenka Seillier, Corinna Lorenz, Katsuhisa Kawaguchi, Torben Ott, Andreas Nieder, Paria Pourriahi, Hendrikje Nienborg

**Affiliations:** ^1^Werner Reichardt Centre for Integrative Neuroscience and; ^2^Department of Animal Physiology, Institute of Neurobiology, University of Tübingen, 72076 Tübingen, Germany

**Keywords:** awake macaque, extracellular recordings, gain modulation, iontophoresis, serotonin, striate cortex

## Abstract

Serotonin, an important neuromodulator in the brain, is implicated in affective and cognitive functions. However, its role even for basic cortical processes is controversial. For example, in the mammalian primary visual cortex (V1), heterogenous serotonergic modulation has been observed in anesthetized animals. Here, we combined extracellular single-unit recordings with iontophoresis in awake animals. We examined the role of serotonin on well-defined tuning properties (orientation, spatial frequency, contrast, and size) in V1 of two male macaque monkeys. We find that in the awake macaque the modulatory effect of serotonin is surprisingly uniform: it causes a mainly multiplicative decrease of the visual responses and a slight increase in the stimulus-selective response latency. Moreover, serotonin neither systematically changes the selectivity or variability of the response, nor the interneuronal correlation unexplained by the stimulus (“noise-correlation”). The modulation by serotonin has qualitative similarities with that for a decrease in stimulus contrast, but differs quantitatively from decreasing contrast. It can be captured by a simple additive change to a threshold-linear spiking nonlinearity. Together, our results show that serotonin is well suited to control the response gain of neurons in V1 depending on the animal's behavioral or motivational context, complementing other known state-dependent gain-control mechanisms.

**SIGNIFICANCE STATEMENT** Serotonin is an important neuromodulator in the brain and a major target for drugs used to treat psychiatric disorders. Nonetheless, surprisingly little is known about how it shapes information processing in sensory areas. Here we examined the serotonergic modulation of visual processing in the primary visual cortex of awake behaving macaque monkeys. We found that serotonin mainly decreased the gain of the visual responses, without systematically changing their selectivity, variability, or covariability. This identifies a simple computational function of serotonin for state-dependent sensory processing, depending on the animal's affective or motivational state.

## Introduction

Perceptually guided behavior depends on context. Such context includes an animal's prior experience or knowledge of the environment and task, and its behavioral and motivational state (Harris and Thiele, 2011). The context dependence of perceptually driven behavior relies, in part, on the context-dependent neuromodulation of sensory processing (Hurley et al., 2004; Harris and Thiele, 2011). One mode of such neuromodulation involves subcortical nuclei that have widespread projections throughout the brain (Jacobs and Azmitia, 1992). These are ideally suited to modulate processing in extended networks according to changing behavioral–motivational conditions (Dayan, 2012).

One important neuromodulatory system is the serotonin system. Serotonin [5-hydroxytryptamine (5-HT)] in the brain has been implicated in a variety of affective, cognitive, and sensorimotor functions, but identifying an account of its computational role even for the intensely studied links to reward signaling has proved challenging (Ranade et al., 2014; Dayan and Huys, 2015). Similarly, the reported effects of serotonin on sensory processing have been heterogeneous (Waterhouse et al., 1990; Hurley et al., 2004; Petzold et al., 2009; Watakabe et al., 2009; Lottem et al., 2016) and a simple computational account remains elusive. For example, in the primary visual cortex (V1) of anesthetized animals' bidirectional modulation of the responses (Watakabe et al., 2009) and variable effects on receptive field properties have been observed (Waterhouse et al., 1990; Hurley et al., 2004; Bachatene et al., 2013). However, despite this multitude of effects on sensory processing, the serotonergic effects on perceptually driven behavior have been surprisingly uniform and consistent with a decreased perceptual response (Davis et al., 1980; Dugué et al., 2014). Potential reasons for these apparent differences are that cortical neuronal modulation in some studies was examined using receptor-selective rather than the endogenous ligand (Watakabe et al., 2009), and these studies were all performed using anesthetized animals. Under anesthesia, substantial fluctuations in brain state that affect responses in the primary sensory cortex have been observed (Ecker et al., 2014). Moreover, anesthesia, such as isofluorane, can directly influence the activity of serotonergic neurons (Johansen et al., 2015). It may therefore be that in awake animals, where the serotonergic system may be in a more controlled state, a less diverse modulation would be seen.

We therefore set out to characterize the modulatory effect of serotonin on sensory processing in the awake animal. To this end, we focused on the macaque V1. The macaque V1 receives extensive projections from the dorsal and medial raphe nuclei, the major source of serotonin in the brain. Serotonergic input projections are most pronounced in the input layers of V1 (de Lima et al., 1988). This pattern is approximately mirrored by the laminar profile of the expression of serotonin receptors in V1 (Watakabe et al., 2009). From a functional perspective, such a bias toward the input layers would be expected, for example, for a mechanism involved in adjusting the gain of the sensory input, as previously suggested (Hurley et al., 2004; Disney et al., 2007). Here, we leveraged the extensive knowledge of the spatiotemporal tuning properties in V1 to quantify the serotonergic modulation on visual processing in awake monkeys along several stimulus dimensions.

Serotonergic neurons are thought to convey a variety of signals on different time scales, as found, for example, for their phasic versus tonic response components (Ranade and Mainen, 2009; Cohen et al., 2015; Fonseca et al., 2015; Hayashi et al., 2015; Correia et al., 2017), versus long-term (days) effects of phasic activation (Correia et al., 2017). In this study we focused on time scales that are consistent with the tonic component of the response by iontophoretically applying serotonin in the minute range time scale.

We find that across the population of neurons in V1 and across different stimulus dimensions, the serotonergic modulation is surprisingly simple: serotonin predominantly decreases the gain of the visual responses, with little change to the tuning properties. A simple additive change to a threshold-linear spiking nonlinearity can account for the observed modulation. Gain modulation is an important computation to change response levels without affecting tuning (Atallah et al., 2012). It has been implicated in the modulation by cognitive states, such as attention (McAdams and Maunsell, 1999), and is subject to cholinergic modulation at the cortical input (Disney et al., 2007). Our results show that serotonin is well suited to control the response gain of neurons in V1, potentially complementing these known gain control mechanisms.

## Materials and Methods

### 

#### 

##### Animals.

Two adult male rhesus monkeys (*Macaca mulatta*; M, 8 kg, 11 years old; K, 12 kg, 7 years old; housed in pairs) participated in the experiments. Using aseptic techniques, the monkeys were implanted with a titanium head-post and titanium chambers over the operculum of V1 under general anesthesia. All experimental procedures followed guidelines for animal experimentation and were approved by the local authorities, the Regierungspräsidium Tübingen, Germany.

##### Electrophysiological recordings and iontophoresis.

We recorded extracellular single-unit activity in V1 while the animals performed a 2 s fixation task (fixation within 0.75° of a small 0.1° fixation dot on the center of the screen) for fluid rewards while we presented stimuli in the receptive field of the recorded unit. The positions of both eyes were recorded at 500 Hz using an infrared optical recording system (Eyelink 1000, SR Research). Experimental control and stimulus presentation was done using custom-written software in Matlab modified after Eastman and Huk (2012) using the psychophysics toolbox (Brainard, 1997; Pelli, 1997; Kleiner et al., 2007).

**Figure 1.**
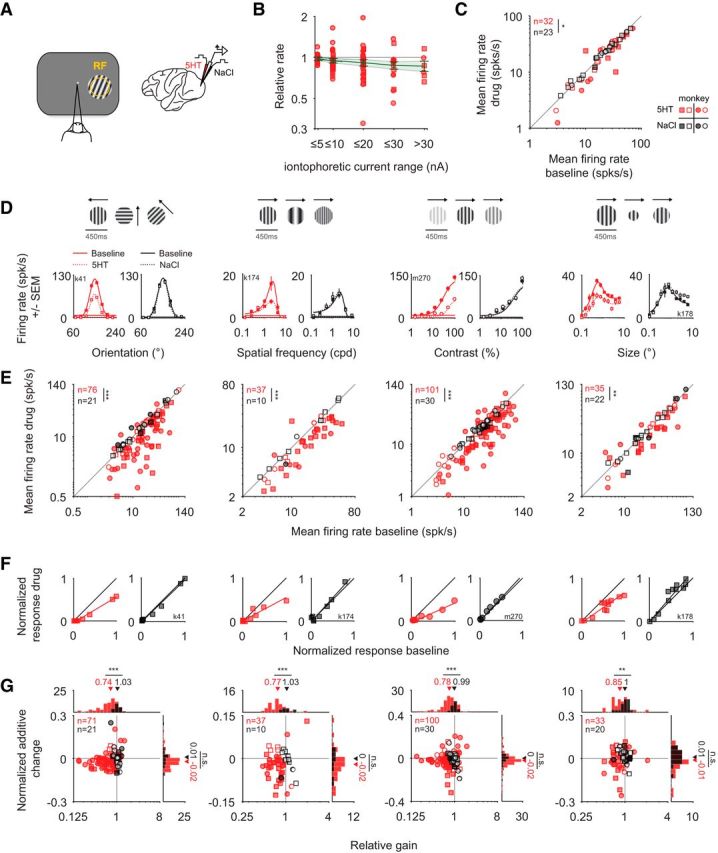
Iontophoretic application of serotonin leads mainly to a gain decrease. ***A***, Schematic of the experimental setup. While the monkey performed a fixation task, grating stimuli were presented in a neuron's receptive field (RF) during blockwise iontophoretic application of serotonin (5HT; red) or pH-matched saline (NaCl; black). ***B***, Dose dependence of the serotonergic modulation. The relative firing rate is plotted as a function of the applied iontophoretic current range of 90 serotonin blocks from *n* = 36 units. Each unit is shown only once per bin. The green line represents the geometric mean ± SEM. ***C***, Modulation for the subset of units for which the duration of unit isolation allowed for a full sequence of baseline, drug application, and recovery. Note that for four units for which tuning for >1 stimulus dimensions was tested, only the first comparison is included. The median of the average firing rate decreased from 27 to 24 spikes/s for serotonin but not for saline application [20 spikes/s for the baseline, 21spikes/s for saline application; *p* = 0.03, *n*_5-HT_ = 32, *n*_NaCl_ = 23; 11 of 32 (34%) of units were significantly suppressed, 2 of 32 (6%) were significantly enhanced for serotonin application; 1 of 23 (4%) was significantly enhanced and suppressed for saline application]. ***D–G***, Orientation, spatial frequency, contrast, and size were varied in blocks, and results are shown in the first, second, third, and last column, respectively. ***D***, Example tuning curves are shown for the baseline condition (red solid line, filled symbols) or the application of serotonin (dotted line, open symbols). Note the decrease in the response amplitude during the application of serotonin. No such change is observed during control experiments when saline is applied instead: the open and closed black symbols largely overlap. ***E***, The mean firing rate (average across the tuning curves as in ***D***, including the blank response) for the baseline condition is compared with that for application of serotonin (red) or saline (black). Note the systematic decrease of the response for serotonin but not for the application of saline. The size of the change in firing rate induced by serotonin or saline, respectively, differs statistically (for orientation: *p* < 10^−5^; for spatial frequency: *p* < 10^−3^; for contrast: *p* < 10^−3^; for size: *p* = 0.001; all Wilcoxon ranked-sum tests). ***F***, To quantify the size of the additive and multiplicative component of the change in the response (normalized to the peak response in the baseline condition), we performed linear (type II) regression on the tuning curves [baseline condition plotted on the abscissa against application of serotonin (red) or saline (black) on the ordinate]. The slope reflects the multiplicative change (relative gain) and the intercept the additive change. ***G***, For each cell, we compared the relative gain and the normalized additive change (normalized by the peak response in the baseline condition). Note that for all stimulus dimensions, the relative gain after applying serotonin is significantly smaller than in the control condition (for orientation: *p* < 10^−5^, *n* = 71 for serotonin; *n* = 21 for saline; for spatial frequency: *p* < 10^−3^; *n* = 37 for serotonin; *n* = 10 for saline; for contrast: *p* < 10^−4^; *n* = 100 for serotonin; *n* = 30 for saline; for size: *p* = 0.003; *n* = 33 for serotonin; *n* = 20 for saline; all Wilcoxon ranked-sum tests). Circles and squares correspond to data from monkey M and K, respectively. Filled symbols represent units with significant mean response modulation (*p* < 0.05, Wilcoxon ranked-sum test). *: <0.05; **:<0.005; ***:<5*10^−4^

Recordings and iontophoresis were done using custom-made tungsten-in-glass electrodes flanked by two pipettes as described previously (Thiele et al., 2006; Jacob et al., 2013). This electrode pipette was mounted inside a guide tube and inserted transdurally without a dura-piercing guide tube using a custom-made electric microdrive. Iontophoretic application was controlled by an MVCS iontophoresis system (NPI Electronic). Neuronal signals were amplified, digitized, and filtered (250 Hz to 5 kHz) with the Ripple Grapevine System (Ripple). Spike sorting was performed off-line using the Plexon Offline Sorter. Spike clusters were computed based on a variety of features, including principal components, energy, peak, trough, and spike amplitude. Single-unit clusters were identified using the features that provided the best separation. Spike isolation was quantified by computing the isolation distance and L ratio (Schmitzer-Torbert et al., 2005). For 763 of 780 (98%) included experimental blocks, the isolation distance was >20 and the L ratio <0.1. For the remaining 17 (2%) experimental blocks that did not meet these criteria, unit isolation was verified by visual inspection. One barrel of the electrode pipette was filled with serotonin hydrochloride (Sigma-Aldrich; 10 mm in double-distilled water; pH 3.5–3.8), the other with pH-matched saline (NaCl; 0.9%). The electrodes typically had impedances between 0.3 and 1.6 MΩ (measured at 1 kHz) and tip sizes of 10–15 μm. The ejection current ranged between 2 and 50 nA (median, 10 nA) for serotonin and between 5 and 20 nA (median, 11 nA) for saline. To better quantify effects on the neuronal tuning properties, we aimed for relatively small modulatory effects. The values of the ejection currents used were therefore toward the lower end of the range of values previously used in the macaque cortex (Williams et al., 2002). In a subset of experiments, we used different ejection currents across blocks to examine the dose dependence of the effect (Fig. 1*B*). We typically used ejection currents less than or equal to the value for which the serotonergic modulation seemed to asymptote (Fig. 1*B*), which we observed at values between 20 and 30 nA, and this limited range of currents may have contributed to the homogeneity of our results. The retention current was −8 nA to prevent leakage from the drug barrels during the control conditions. The pipette resistance ranged from 10 to 150 MΩ, as was used previously (Ott et al., 2014). To minimize long-term effects of serotonin (Maya Vetencourt et al., 2008; Correia et al., 2017), we avoided recording from nearby locations in V1 in consecutive recording sessions.

For each unit we initially quantified the center of the receptive field from receptive-field profiles along a horizontal and vertical axis as described previously (Nienborg et al., 2004) by presenting an elongated rectangular grating (height, 3–5°; width, 0.2°) at different horizontal or vertical positions across the receptive field and its immediate surroundings. Subsequent stimuli were centered on the receptive field at a median eccentricity of 3.6° (range, 1.6 to 6.4°). We initially measured neuronal tuning curves without applying serotonin or saline to establish the baseline using the retention current. We then started to apply serotonin or saline using the ejection current in blocks and then remeasured a tuning curve during the application (note that our analyses of the time course of the response modulation across trials averaged over all experiments did not reveal a systematic difference between the baseline, saline control, and serotonin experiments, suggesting that the lead time of the serotonin application of several seconds was, on average, sufficient). Following the application of the drug, we remeasured the tuning in subsequent blocks using the retention current to obtain the recovery of the response if it was possible to maintain unit isolation. Thus, if it was possible to maintain unit isolation and keep the monkey working, each block of drug application was followed by ≥1 block of recovery before the next application of the drug. Full recovery was evaluated by observation during recording but verified statistically off-line: the responses to each stimulus parameter were *z*-scored to remove the stimulus-driven variability of the response, and the *z* scores for the baseline and recovery block were then required to be statistically indistinguishable (*p* > 0.05, Wilcoxon rank-sum test). The median time to achieve full recovery [possible for *n* = 90 serotonin experiments for which the inclusion criteria (see below) were met; this value includes multiple experiments per unit, e.g., for our dose–response measurements; Fig. 1*B*] after the serotonin application was 32 s (range, 15 to 1171 s). Note that these values reflect the upper bound of the times to full recovery since we occasionally inserted wait times of variable duration after the serotonin application experiment to ensure full recovery while keeping the animal motivated to perform additional trials afterward.

##### Stimuli.

Visual stimuli were back-projected on a screen using DLP LED Propixx projector (1920 × 1080 pixels resolution; 30 cd/m^2^ mean luminance; linearized gray values; run at 100 Hz/eye, combined with an active circular polarizer, DepthQ, run at 200 Hz) at a distance of 98 cm in front of the animals. The animals viewed the screen through passive circular polarizing filters monocularly or binocularly. Visual stimuli were generated in Matlab (Mathworks) using the Psychophysics toolbox (Brainard, 1997; Pelli, 1997; Kleiner et al., 2007).

Stimuli were circular drifting sinusoidal luminance gratings centered on and slightly exceeding a neuron's receptive field and presented for 450 ms (temporal frequency typically 7 Hz) binocularly or monocularly to the preferred eye. For each experimental block, either the direction (16 equally spaced values), the spatial frequency [eight logarithmically spaced values from 0.125 to 16 cycles per degree (cpd)], the contrast (typically seven logarithmically spaced values from 1.56 to 100%), or the size (typically 12 logarithmically spaced values from 0.3 to 8°) of the grating was pseudorandomly varied, randomly interleaved by blank stimuli, with all other parameters constant at approximately the preferred value for each unit. Within each experimental block each stimulus was typically presented 8–10 times.

For the subspace reverse correlation, we briefly flashed sinusoidal luminance gratings (flash duration typically 10 ms; 100% contrast; preferred spatial frequency; presented binocularly or monocularly to the preferred eye) that randomly varied in orientation (eight equally spaced values) and spatial phase (equally spaced values), randomly interleaved by blank stimuli (also 10 ms duration, presented with equal probability as each orientation).

##### Data analysis.

All analyses were done in Matlab (Mathworks). For experiments using drifting gratings, we computed the mean firing rate during the 450 ms stimulus presentation. To obtain orientation tuning curves, we averaged the responses for directions 180° apart. Tuning curves were computed as the mean firing rate as a function of orientation, spatial frequency, contrast, and size, respectively, and fit with standard descriptive functions.

Orientation tuning curves were fit with Gaussian functions for which all parameters were constrained to values >0. To avoid an overestimation of the amplitude resulting from placing the preferred orientation between two sparsely measured values, we further restricted the amplitude to be smaller than twice the peak value of the tuning curve.

Spatial frequency tuning curves were also fit with Gaussian functions, either in linear or logarithmic units, whichever resulted in better fits.

Contrast tuning curves were fit with the Naka–Rushton function (Albrecht and Hamilton, 1982): *R*(*c*) = *R*_max_ *c^n^*/(*c*_50_^*n*^ + *c^n^*) + *R*_offset_, where *c* is the stimulus contrast, *c*_50_ is the semisaturation contrast, and *n* is the exponent influencing the shape of the curve. All parameters were restricted to values >0, and additionally *R*_max_ + *R*_offset_ could not exceed the peak response of the tuning curve to ensure that fits reached saturation within the contrast range of 0 to 100%. For the model comparison in Figure 6, we first fit the Naka–Rushton function to the baseline condition. In a second step, to account for the serotonin-induced modulation of the response, we allowed one parameter of this fit to the baseline to change: *R*_max_ for the response-gain model, or *c*_50_ for the contrast-gain model (Williford and Maunsell, 2006).

Size tuning curves were fit with a ratio-of-Gaussians function (Cavanaugh et al., 2002). All parameters had to be positive, and the width of the center Gaussian had to be less than or equal to that of the surround Gaussian function. The preferred size was defined as the smallest size that evoked 98% of the maximal response based on the fitted data (Nienborg et al., 2013). The suppression index was computed as the difference between the neuron's maximum response and the response to the maximum size, divided by the maximum response. Goodness of fit was quantified as variance explained and all fits had to explain ≥70% of the variance.

For a subset of units we also measured temporal frequency tuning with stimulus presentations of 2 s each. We used these longer stimulus presentations to quantify the selectivity to spatial phase for each unit as the *f*1/*f*0 ratio (Skottun et al., 1991) measured at the preferred temporal frequency of that unit, where *f*1 corresponds to the amplitude of response modulation at the stimulus temporal frequency and *f*0 to the mean firing rate. For these analyses, eye movements within the fixation window were not factored out, which may have contributed to the modest phase selectivity across the population.

Orientation selectivity was quantified using the circular variance (Ringach et al., 2002). Direction selectivity was computed as a simple contrast metric (*r*_θ_−*r*_θ−180_)/(*r*_θ_ + *r*_θ−180_), where *r*_θ and_ *r*_θ−180_ correspond to the response at the neuron's preferred direction θ and that at 180° away from preferred, respectively.

Receptive field size was quantified as the mean of the equivalent widths (*w*; Bracewell, 1986) in both the vertical and horizontal directions: If *A* is the area under the horizontal or vertical receptive field profile, and *h* is the peak response of the receptive field profile, then *w* = *A*/*h*. The median equivalent width was 0.56° (range, 0.25 to 1.34°).

To evaluate significant response modulation during the serotonin or saline condition, we *z*-transformed the mean response for each stimulus condition and compared the *z* scores across all stimuli between the serotonin or saline condition and the baseline condition using Wilcoxon rank-sum test (two-sided, 5% significance threshold).

For the experiments with flashed gratings, we quantified the tuning curves using reverse correlation subspace analysis (Ringach et al., 1997). We smoothed the stimulus-triggered spike-density function (SDF) using a 4 ms boxcar. The SDFs were used to compute the mean number of spikes elicited by each frame to yield “orientation subspace maps,” analogous to an approach described previously in the disparity domain (Nienborg and Cumming, 2009). SEs were estimated based on bootstrapping (1000 resamples). To estimate the dynamics of the orientation-selective component of the response, we first computed the SD across the SDFs for each orientation. Response latencies were defined as the first point in time after frame onset for which the SD exceeded the half-height between the baseline variability and peak deviation (Lee et al., 2007). The baseline variability was computed as the average of the first 20 ms after frame onset. For inclusion of latency estimates, the orientation-selective response component had to exceed four times the baseline variability. Additionally, latencies were restricted to 25–100 ms after frame onset. To ensure that changes in latency could not be explained by differences in the response variability between conditions, we estimated the dependence of the latency estimate on baseline variability by randomly subsampling the data in the baseline condition and computing latency for the subsampled data. The change in latency was then predicted from the change in baseline variability using linear regression, and the actually measured latency was corrected by the predicted change (see Fig. 5*D*). For comparison, we also quantified response latency using maximum likelihood estimation to identify the critical change point of the dynamics of the response (Friedman and Priebe, 1998). This approach assumes one Poisson process for the baseline response and one for the stimulus-evoked component of the response, and we required significantly different Poisson processes for inclusion. This analysis yielded similar results.

##### Analysis of fixational eye movements.

To examine potential effects on fixational eye movements, we explored the fixation precision as well as the amplitude and frequency of microsaccades. Microsaccades within the fixation window were identified as defined previously (Nienborg and Cumming, 2006, 2014; Clery et al., 2017). Fixation precision was defined by Cherici et al. (2012) as the area around the mean gaze position that encompassed the 75^th^ percentile of the gaze positions.

##### Noise correlations.

Stimulus-independent covariability [“noise correlations,” (Cohen and Kohn, 2011)] was calculated between the single-unit activity and the simultaneously measured multiunit activity recorded from the same electrode. Note that since this approach overestimates the absolute value of noise correlation (Ecker et al., 2010), we only explored the changes in noise correlation with serotonin, not the absolute value. To reduce the effect of slow fluctuations on nonstationarities (Ecker et al., 2014; Goris et al., 2014; Rabinowitz et al., 2015) resulting from the onset of the serotonin application, we removed the initial 20 stimulus presentations of each experimental block for this analysis. Responses for each stimulus condition were first *z*-scored, and noise correlations computed as the Pearson correlation coefficient of the *z* scores for single-unit and multiunit activity across all trials. Noise correlations have typically been found to depend on the neuronal spike rate (Cohen and Kohn, 2011). We estimated this dependence in our dataset for the baseline condition by linear regression and corrected the noise correlation by that predicted from the change in firing rate.

##### Fano factor.

For each stimulus condition, we computed the Fano factor as the ratio between the variance and the mean response for each stimulus for each experimental block, excluding the initial 20 stimulus presentations to reduce variability from potential nonstationarities due to onset of serotonin. The average of the Fano factors for each stimulus was then calculated as the Fano factor for that unit.

##### Membrane potential-based model.

The responses of the membrane potential-based model were explored for the stimulus used for orientation subspace mapping described earlier. The membrane potential was orientation selective and the selectivity described by a Gaussian function [amplitude, 20 mV; similar to empirically observed values (Priebe and Ferster, 2008)]. The width of the Gaussian function describing the orientation selectivity of the membrane potential (SD ranged from 10 to 40°) was chosen such that the bandwidth of the orientation subspace maps was within the range of those of the neuronal data in the baseline condition. The time-varying stimulus (a sequence of oriented gratings each flashed for 10 ms) induced fluctuations in membrane potential *V_m_*(*t*) that were convolved by a temporal kernel that consisted of a Gaussian temporal filter (chosen to be within the range of the neuronal data: SD, 6 ms; mean, 57 ms after stimulus onset to account for the lag of the response). Spike rates [*k*(*t*)] were derived from the following threshold-linear function:

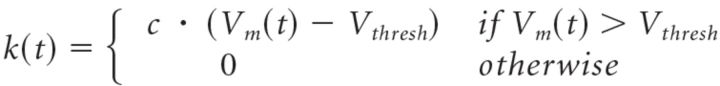
 where *V_m_*(*t*) denotes the membrane potential at time *t*, *V*_thresh_ is the spiking threshold, and *c* is a scalar value set to 15, approximating the value previously obtained for the cat striate cortex (Carandini, 2004). The spiking rate was converted to Poisson spike events, which were analyzed like the neuronal data.

The serotonergic modulation was imitated by a subtractive shift in the membrane potential (equivalent to changing *V*_thresh_). We explored shifts over a range of 1 to 8 mV, similar to empirically observed changes in membrane potential in response to serotonin (Ko et al., 2016). We adjusted the number of stimulus repetitions to obtain a comparable level of baseline variability for the baseline and serotonin condition.

##### Inclusion criteria.

**Figure 2.**
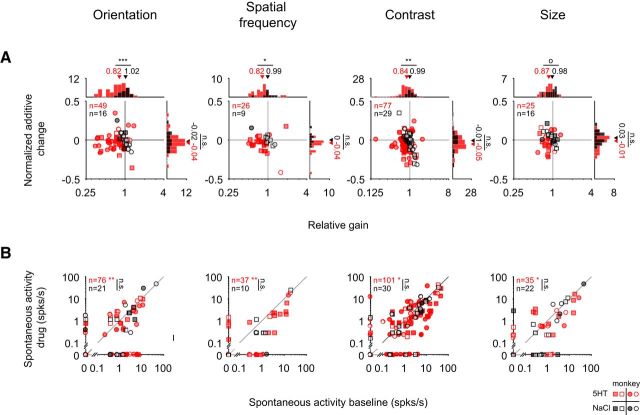
The gain decrease with serotonin does not merely reflect the iceberg effect. Symbols are the same as in Figure 1. Note that filled symbols reflect units with significant response modulation based on the full tuning curves as in Figure 1*E*. ***A***, Comparing the gain and additive changes to the tuning curves as in Figure 1*G* but after restricting the datapoints to those whose responses were >0 spikes/s to explore the serotonergic modulation independent of the iceberg effect. In this reduced dataset, we also observe a significant gain decrease (relative gain for orientation: 0.82 for serotonin and 1.02 for saline, *p* < 10^−3^, *n*_5-HT_ = 49, *n*_NaCl_ = 16; for spatial frequency: 0.82 for serotonin and 0.99 for saline, *p* = 0.016, *n*_5-HT_ = 26, *n*_NaCl_ = 9; for contrast: 0.84 for serotonin, 0.99 for saline, *p* = 0.003, *n*_5-HT_ = 77, *n*_NaCl_ = 29; for size: 0.87 for serotonin, 0.98 for saline, *p* = 0.075, *n*_5-HT_ = 25, *n*_NaCl_ = 16). ***B***, The application of serotonin mainly resulted in a decrease of the blank response (blank interleaved in orientation (*p*_5-HT_ = 0.005, *n*_5-HT_ = 76; *p*_NaCl_ = 0.35, *n*_NaCl_ = 21), spatial frequency (*p*_5-HT_ = 0.001, *n*_5-HT_ = 37; *p*_NaCl_ = 0.58, *n*_NaCl_ = 10), contrast (*p*_5-HT_ = 0.02, *n*_5-HT_ = 101; *p*_NaCl_ = 0.09, *n*_NaCl_ = 30), or size (*p*_5-HT_ = 0.03, *n*_5-HT_ = 35, *p*_NaCl_ = 0.50, *p*_NaCl_ = 22) experiment (all Wilcoxon paired signed-rank tests).

For each unit, we required a minimum response to the neuron's preferred stimulus of 10 spikes/s, a minimum of four presentations per stimulus condition (except for the noise correlation and Fano factor analysis, where ≥8 presentations per stimulus condition were required), and that the neuron showed selectivity for the respective stimulus dimension (orientation, spatial frequency, contrast, size; evaluated by an ANOVA at a significance threshold of *p* < 0.01). To be included in the comparison of additive and multiplicative changes (Figs. 1, 2, 5), the type-II regression had to account for ≥70% of the variance.

## Results

Two macaque monkeys performed a standard fixation task while we recorded the activity of single units in their V1 during blockwise iontophoretic application of serotonin (Fig. 1*A*). We examined the effect of serotonin on the visual responses to drifting gratings that varied systematically in orientation, spatial frequency, contrast, or size, and to briefly flashed gratings of varying orientation (see Materials and Methods). We recorded a total of 265 single units in macaque V1 (118 from monkey M, and 147 from monkey K). To be included for further analysis, we required a minimum response to the neuron's preferred stimulus of 10 spikes/s, ≥4 presentations per stimulus condition, and that the neuron showed selectivity for the respective stimulus dimension as evaluated by an ANOVA at a significance threshold of *p* < 0.01. These criteria were passed by 229 units (108 for monkey M; 121 for monkey K). Of these, 206 (100 for monkey M; 106 for monkey K) were recorded with serotonin application and 65 (39 for monkey M; 26 for monkey K) with pH-matched saline (NaCl) application as control experiments (thus, in a subset of 42 units, we were able to measure responses for both serotonin and saline application in consecutive blocks; moreover, whenever possible, experiments along several stimulus dimensions were done on the same unit in consecutive blocks).

### Serotonin predominantly decreases the responses in V1 by multiplicative changes of the tuning curves

The most salient consequence of the serotonin application was a substantial decrease in the visual responses. This effect was evident for the tuning curves for orientation, spatial frequency, contrast, and size, respectively, in four example neurons (Fig. 1*D*, red symbols). We found that these response changes could not be explained by the iontophoretic current application: control experiments with pH-matched saline (NaCl) did not result in such modulation of the neuronal responses (Fig. 1*D*, black symbols). Indeed, across the population, the mean firing rate in response to gratings of different orientations decreased for the serotonin condition (the median decrease was from 18 to 10 spikes/s) but not for the saline application (median values, 14 and 15 spikes/s, respectively), and the changes differed significantly between conditions [Fig. 1*E*; *p* < 10^−5^, *n*_5-HT_ = 76, *n*_NaCl_ = 21; monkey M: *p* < 10^−3^, *n*_5-HT_ = 45, *n*_NaCl_ = 11; monkey K: *p* < 10^−3^, *n*_5-HT_ = 31, *n*_NaCl_ = 10; significant modulation for serotonin: 51 of 76 units (67%) were suppressed; 4 of 76 units (5%) were enhanced; significant modulation for saline: 4 of 21 units (19%) were suppressed; 3 of 21 units (14%) were enhanced]. These results were similar for gratings of varying spatial frequency, contrast, and size (Fig. 1*E*). For spatial frequency, the median average firing rate for serotonin decreased from 15 to 12 spikes/s and remained constant at 11 spikes/s for saline application [*p* < 10^−3^, *n*_5-HT_ = 37, *n*_NaCl_ = 10; monkey M: *p* = 0.41, *n*_5-HT_ = 11, *n*_NaCl_ = 2; monkey K: *p* = 0.001, *n*_5-HT_ = 26, *n*_NaCl_ = 8; significant modulation for serotonin: 24 of 37 units (65%) were suppressed, 2 of 37 units (5%) were enhanced; significant modulation for saline: 1 of 10 units (10%) was suppressed]. For contrast tuning, the median firing rate decreased from 26 to 19 spikes/s for serotonin [the respective values for saline application were 23 and 22 spikes/s; *p* < 10^−3^, *n*_5-HT_ = 101, *n*_NaCl_ = 30; monkey M: *p* = 0.04, *n*_5-HT_ = 66, *n*_NaCl_ = 15; monkey K: *p* = 0.004, *n*_5-HT_ = 35, *n*_NaCl_ = 15; significant modulation for serotonin: 58 of 101 units (57%) were suppressed, 8 of 101 (8%) units were enhanced; significant modulation for saline: 4 of 30 (13%) were suppressed]. Similarly, the mean firing rate decreased for the size tuning curves [29 to 25 spikes/s for serotonin; constant at 18 spikes/s for saline, *p* = 0.001, *n*_5-HT_ = 35, *n*_NaCl_ = 22; monkey M: *p* = 0.65, *n*_5-HT_ = 14, *n*_NaCl_ = 6; monkey K: *p* < 10^−4^, *n*_5-HT_ = 21, *n*_NaCl_ = 16; all Wilcoxon rank-sum tests; significant modulation for serotonin: 19 of 35 (54%) were suppressed, 5 of 35 (14%) were enhanced; significant modulation for saline: 2 of 22 (9%) were suppressed, 5 of 22 (23%) were enhanced].

In a subset of experiments, unit isolation was maintained and the animal worked for sufficiently long for a full sequence of baseline, drug application, and recovery. The results for this subset of experiments are shown in Figure 1*C* (same format as Fig. 1*E*), also supporting the significant decrease in firing when serotonin was applied. The proportion of units for which full, statistically defined (see Materials and Methods), recovery was achieved was relatively small mainly because of sessions in which the monkeys were not motivated to continue to work or the unit isolation was lost. However, this relatively small proportion does not affect the interpretation of the main results of this paper, which relies on a comparison of the serotonergic modulation with that for pH-matched saline, and this comparison was robust when restricting the data to the subset of units with full recovery and saline control experiments (Fig. 1*C*). To additionally verify that a lack of recovery for some serotonin experiments did not impact the saline control experiments, we also performed two additional analyses. First, we compared the serotonergic modulation with that for saline for units for which only one substance (either serotonin or saline) per unit was applied. Second, we compared the serotonergic modulation for units with full recovery with the subset of the same dataset for which we also applied saline. For both analyses, we found a significant difference between the modulation for serotonin and that for saline (comparison across units: *p* < 10^−7^, *n*_5-HT_ = 53, *n*_NaCl_ = 23 and comparison for units with full recovery: *p* = 0.04, *n*_5-HT_ = 32, n_NaCl_ = 11, respectively; note that for each unit only the first experiment per condition was included; Wilcoxon rank-sum tests). Moreover, our analysis of the responses to the interleaved blank stimuli revealed that the change in firing was not limited to the stimulus-driven response, but also observed in response to the blank stimuli (Fig. 2*B*).

For the example tuning curves (Fig. 1*D*) serotonin seems to primarily scale down the tuning curve, indicative of a gain decrease (multiplicative effect). We then quantified across the population whether the effect of serotonin was primarily additive or multiplicative. To do so, we compared the tuning curves with and without serotonin application, fit by type-II regression (Fig. 1*F*). The slope and intercept of the regression line reflect the multiplicative (relative gain) and additive change. When comparing these values for each unit across the population, we found that the changes in the tuning curve were primarily accounted for by a gain decrease for each type of tuning curve. For orientation tuning curves, the median gain decreased to 74% after serotonin application, in contrast to saline (median relative gain for saline: 103%; *p* < 10^−5^, *n*_5-HT_ = 71, *n*_NaCl_ = 21; monkey M: *p* < 0.01, *n*_5-HT_ = 43, *n*_NaCl_ = 11; monkey K: *p* < 10^−4^, *n*_5-HT_ = 28, *n*_NaCl_ = 10; Fig. 1*G*). Similarly, for spatial frequency, the gain reduced on average to 77% for serotonin (median relative gain for saline: 103%; *p* < 10^−3^, *n*_5-HT_ = 37, n_NaCl_ = 10; monkey M: *p* = 0.23, *n*_5-HT_ = 11, *n*_NaCl_ = 2; monkey K: *p* < 10^−3^, *n*_5-HT_ = 26, *n*_NaCl_ = 8). For contrast tuning, the gain decreased on average to 78% for serotonin (for saline the median relative gain was 99%; *p* < 10^−4^, *n*_5-HT_ = 100, *n*_NaCl_ = 30; monkey M: *p* < 0.01, *n*_5-HT_ = 66, *n*_NaCl_ = 15; monkey K: *p* < 10^−4^, *n*_5-HT_ = 34, n_NaCl_ = 15). As for the other stimulus dimensions, the median gain for size tuning was a decrease (to 85%) for serotonin while for saline the median relative gain was unchanged at 100% (*p* = 0.003, *n*_5-HT_ = 33, *n*_NaCl_ = 20; monkey M: *p* = 0.78, *n*_5-HT_ = 13, *n*_NaCl_ = 5; monkey K: *p* < 10^−3^, *n*_5-HT_ = 20, *n*_NaCl_ = 15; all comparisons Wilcoxon rank-sum tests). The normalized additive suppressive effect differed significantly from 0 for the serotonin but not for the saline conditions for orientation, spatial frequency, and contrast (for orientation: −0.02 for serotonin, *p* < 10^−3^, *n* = 76; 0.01 for saline, *p* = 0.96, *n* = 21; for spatial frequency: median normalized additive change for serotonin was −0.02, *p* < 0.01, *n* = 37; change for saline was 0.00, *p* = 0.56, *n* = 10; for contrast the median normalized additive change for serotonin was −0.02, *p* < 10^−3^, *n* = 100 and 0.00 for saline, *p* = 0.45, *n* = 30; for size: the corresponding changes were −0.01 for serotonin, *p* = 0.62, *n* = 33, and 0.01 for saline, *p* = 0.26, *n* = 20; Wilcoxon signed-rank test) but given the variability in the control condition, the additive suppression for serotonin did not significantly exceed that for the control condition (for orientation: *p* = 0.12, *n*_5-HT_ = 71, *n*_NaCl_ = 21; for spatial frequency: *p* = 0.29, *n*_5-HT_ = 37, *n*_NaCl_ = 10; for contrast: *p* = 0.21, *n*_5-HT_ = 100, *n*_NaCl_ = 30; for size: *p* = 0.20, *n*_5-HT_ = 33, *n*_NaCl_ = 20; Wilcoxon rank-sum test). Moreover, the absence of an additive suppressive effect seemed to at least partially result from the “iceberg effect” (Carandini and Ferster, 2000): as a consequence of the spiking nonlinearity, spike rates are restricted to values >0 such that subtractive changes are limited by the minimum response of the tuning curve. In support of this, we found a significant correlation between the size of the normalized additive change and the minimum response of the tuning curve for the baseline condition (*r* = −0.41, *p* < 10^−8^, *n*_5-HT_ = 198; Spearman's rank correlation; for this comparison we combined data for different stimulus dimensions but included only one data point per unit; data not shown). To verify that the observed gain decrease was not merely a consequence of the iceberg effect, we repeated the regression analysis after removing all data for which the responses were 0. In the subset of units for which the regression fits to the reduced dataset met the inclusion criteria (see Materials and Methods), we also found a significant gain decrease (Fig. 2*A*). Together, the additive and multiplicative changes provided an excellent fit to the data. Indeed, this simple linear model accounted for 90% of the variance (averaged across all regression fits for serotonin in Fig. 1*G*; note that the quality of the fits was similar for the saline controls: 92%). These results suggest that a simple linear transformation, predominantly multiplicative, can account for the serotonergic modulation of visual responses along several visual dimensions.

**Figure 3.**
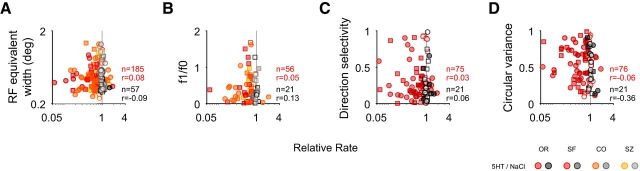
Probing the serotonergic modulation as a function of receptive field (RF) size and phase, direction, and orientation selectivity. ***A–D***, Symbols are the same as in Figure 1. Shading represents the type of experiment for which the modulation by drug application [serotonin (5HT) or saline (NaCl)] was quantified. The modulation was quantified as the relative rate based on the average firing rates across the tuning curve (compare Fig. 1*E*) for the drug condition (compare Fig. 1*E*, ordinate), divided by that for the baseline condition (compare Fig. 1*E*, abscissa). Neither RF size, quantified as equivalent width (***A***); phase selectivity, quantified as f1/f0 (***B***); direction selectivity (***C***); nor orientation selectivity, quantified as circular variance (***D***), were correlated with the modulation by serotonin or saline. Note that low values for circular variance reflect strong orientation selectivity (all Spearman's rank correlation).

Given that serotonin receptors are differentially expressed across layers and on different cell types in the macaque V1 (Watakabe et al., 2009) the overall homogeneity of the effect is surprising. We therefore wondered whether the serotonergic modulation was systematically related to parameters that have been reported to vary to a certain degree with layer, such as receptive field size, orientation, direction, and spatial-phase selectivity (Ringach et al., 2002; Gur et al., 2005). To test this, we compared each of these parameters with the strength of serotonergic modulation (Fig. 3). Our analyses revealed no correlation of the serotonergic modulation with these parameters (*r* = 0.08, *p* = 0.28, *n* = 185 for equivalent width; *r* = 0.05, *p* = 0.72, *n* = 56 for *f*1/*f*0 ratio; *r* = 0.03, *p* = 0.77, *n* = 75 for direction tuning index; *r* = −0.06, *p* = 0.62, *n* = 76 for circular variance; Spearman's rank correlation with serotonergic modulation ratio for each). Moreover, we did not observe a systematic difference in the serotonergic modulation for narrow spiking compared with broad spiking (defined by a spike waveform width of <200 or ≥200 μs, respectively) units (*p* = 0.49; *n* = 206; Wilcoxon rank-sum test). Together, these analyses suggest that the observed serotonergic modulation was not restricted to a particular cell type or layer.

### Serotonin leaves visual tuning properties largely unchanged

**Figure 4.**
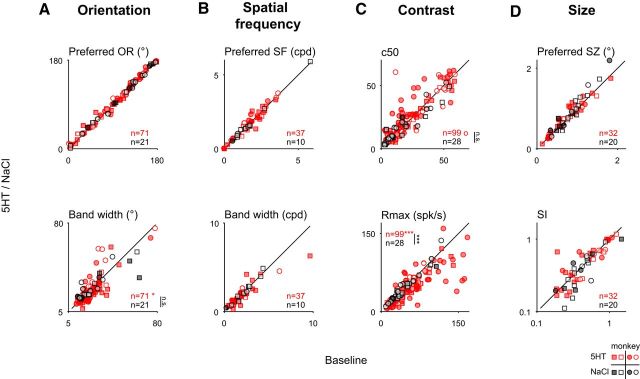
Serotonin does not systematically change the neuronal selectivity. Filled symbols reflect units with significant response modulation based on the full tuning curves as in Figure 1*E*. ***A–D***, The neuronal tuning curves for orientation (***A***), spatial frequency (***B***), contrast (***C***), and size (***D***) were fit with descriptive functions (see Materials and Methods) to quantify changes in selectivity. Symbols are the same as in Figure 1. ***A***, For orientation tuning, we compared the mean (preferred orientation, top) and the SD (orientation tuning width, bottom) of Gaussian fits. There was no significant change for either parameter that exceeded changes observed for saline (preferred orientation: *p* = 0.29, *n* = 71 for serotonin; *p* = 0.66, *n* = 21 for saline; bandwidth: *p* = 0.05, *n* = 71 for serotonin; *p* = 0.93, *n* = 21 for saline; *p* = 0.24 for the Wilcoxon ranked-sum test). ***B***, For the spatial frequency tuning curves, neither the mean (preferred spatial frequency, top) nor the SD (bandwidth, bottom) of the fitted Gaussians differed significantly (mean: *p* = 0.50, *n* = 37 for serotonin; *p* = 0.92, *n* = 10 for saline; SD: *p* = 0.11, *n* = 37 for serotonin; *p* = 0.85, *n* = 10 for saline). ***C***, For the contrast tuning, the contrast at half-maximum showed a nonsignificant trend toward higher values (top; *C*_50_; *p* = 0.06, *n* = 99 for serotonin; *p* = 1.0, *n* = 28 for saline). The maximum response (*R*_max_, bottom) decreased significantly for serotonin, as expected for a gain decrease. Indeed, the change in the maximum response differed significantly between the serotonin and the saline conditions (*p* < 10^−4^, *n* = 99 for serotonin, *n* = 28 for saline; Wilcoxon ranked-sum test). ***D***, For size tuning, we found no significant difference in the preferred size (top; *p* = 0.70, *n* = 32 for serotonin; *p* = 0.09, *n* = 20 for saline) or surround index (bottom; *p* = 0.54, *n* = 32 for serotonin; *p* = 0.30, *n* = 20 for saline). All paired comparisons used the Wilcoxon paired signed-rank test.

We next wondered whether the serotonin application additionally resulted in a systematic modulation of the visual encoding properties along one of the visual stimulus dimensions we explored. To this end we fit descriptive functions (see Materials and Methods) to the tuning curves and examined the effect of serotonin parametrized by these fits. For orientation tuning, the serotonergic modulation changed neither the preferred orientation (Fig. 4*A*, top; *p* = 0.29, *n*_5-HT_ = 71; monkey M: *p* = 0.34, *n*_5-HT_ = 41; monkey K: *p* = 0.63, *n*_5-HT_ = 30) nor the orientation bandwidth (Fig. 4*A*, bottom; *p* = 0.05, *n*_5-HT_ = 71; monkey M: *p* = 0.18, *n*_5-HT_ = 41; monkey K: *p* = 0.23, *n*_5-HT_ = 30; Wilcoxon paired sign-rank tests; note that the slight decrease in bandwidth did not significantly differ from the saline condition, *p* = 0.24, *n*_NaCl_ = 21). Similarly, serotonin did not alter the preferred spatial frequency (Fig. 4*B*, top; *p* = 0.50, *n*_5-HT_ = 37; monkey M: *p* = 0.32, *n*_5-HT_ = 11; monkey K: *p* = 0.12, *n*_5-HT_ = 26) or spatial frequency bandwidth of the recorded neurons (Fig. 4*B*, bottom; *p* = 0.11, *n*_5-HT_ = 37; monkey M: *p* = 0.97, *n*_5-HT_ = 11; monkey K: *p* = 0.11, *n*_5-HT_ = 26; Wilcoxon paired sign-rank tests). However, the amplitude of the fits was significantly reduced for both orientation (*p* < 10^−4^, *n*_5-HT_ = 71, *n*_NaCl_ = 21; monkey M: *p* < 0.01, *n*_5-HT_ = 41, *n*_NaCl_ = 11; monkey K: *p* < 0.01, *n*_5-HT_ = 30, *n*_NaCl_ = 10) and spatial frequency (*p* < 0.01, *n*_5-HT_ = 37, *n*_NaCl_ = 10; monkey M: *p* = 0.41, *n*_5-HT_ = 11, *n*_NaCl_ = 2; monkey K: *p* < 0.01, *n*_5-HT_ = 26, *n*_NaCl_ = 8; all Wilcoxon sign-rank tests), as expected given the observed reduction in gain. Also as expected from the reduction in gain, we observed a significant reduction in *R*_max_ for contrast tuning (Fig. 4*C*, bottom; *p* < 10^−4^, *n*_5-HT_ = 99, *n*_NaCl_ = 28; monkey M: *p* < 0.01, *n*_5-HT_ = 66, *n*_NaCl_ = 14; monkey K: *p* < 10^−3^, *n*_5-HT_ = 33, *n*_NaCl_ = 14; Wilcoxon sign-rank test). Conversely, the sensitivity for changes in contrast (i.e., the steepness of the tuning curve parametrized by the exponent *n* in the Naka–Rushton function; see Materials and Methods), did not change (*p* = 0.15, *n* = 99; monkey M: *p* = 0.43, *n* = 66; monkey K: *p* = 0.19, *n* = 33; Wilcoxon paired sign-rank test; data not shown). Interestingly, we also found that the contrast that yielded half the maximum response (c_50_) was slightly increased. We note that this trend did not reach statistical significance in the population and was only significant in one of the animals (*p* = 0.06, *n*_5-HT_ = 99; monkey M: *p* = 0.01, *n*_5-HT_ = 66; monkey K: *p* = 0.56, *n*_5-HT_ = 33). Nonetheless, this trend raises the question whether serotonin engages a similar mechanism as contrast gain control, which we will address in more detail below.

Finally, we examined whether serotonin had a systematic effect on receptive field size, as suggested by previous work in anesthetized rats (Waterhouse et al., 1990). To address this question, we first compared the stimulus size for which the visual response was maximal (“preferred size”) with and without application of serotonin. In contrast to the previous suggestion, we found no systematic change in the preferred size of the neurons (Fig. 4*D*, top; *p* = 0.70, *n*_5-HT_ = 32; monkey M: *p* = 0.41, *n*_5-HT_ = 12; monkey K: *p* = 0.30, *n*_5-HT_ = 20; Wilcoxon paired sign-rank tests). To explore the effect of center surround interactions, we also examined the degree to which the visual responses to large stimuli decreased the visual stimuli compared with stimuli of a neuron's preferred size (“suppression index”). Similar to the results for preferred size, we found no significant effect of serotonin on the neurons' surround suppression quantified by the suppression index (Fig. 4*D*, bottom; *p* = 0.54, *n*_5-HT_ = 32; monkey M: *p* = 0.38, *n*_5-HT_ = 12; monkey K: *p* = 0.74, *n*_5-HT_ = 20; Wilcoxon paired signed-rank tests).

Together these analyses corroborate our finding that the observed serotonergic modulation is dominated by a multiplicative change and modest additive change of the visual responses. Beyond that, serotonin leaves the receptive field properties largely unchanged.

### Serotonin weakly increases the latency of the orientation selective response

**Figure 5.**
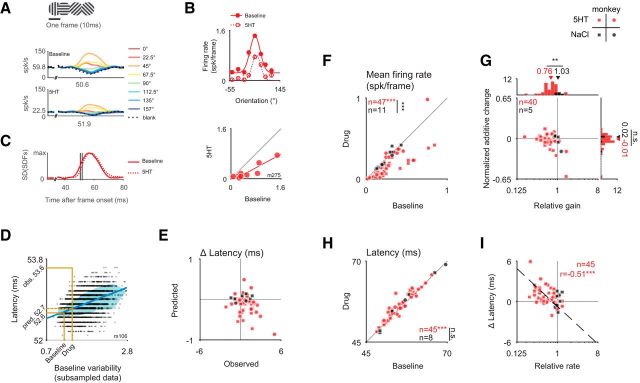
A slight modulation of the response dynamics by serotonin. Symbols are the same as in Figure 1. ***A***, SDFs separated by orientation for one example neuron without (top) and with (bottom) application of serotonin obtained for random sequences of briefly (10 ms) flashed gratings of different orientations. ***B***, The serotonergic application for this unit resulted in both a modest subtractive change and a divisive change of the tuning curve. ***C***, To quantify the dynamics of the response, we computed the SD across the SDFs for each orientation. These SDs are superimposed for the baseline (solid) and serotonin condition. Latency was defined as the time to the half-maximum response. Note the slight increase in latency for the serotonin condition. ***D***, In control analyses, we subsampled the data of the baseline condition to quantify changes in latency estimates due to changes in baseline variability. ***E***, The predicted changes in latency from changes in baseline variability cannot account for the observed latency changes. Note the different scales of abscissa and ordinate. ***F***, The mean firing rate for the orientation subspace stimulus decreases significantly with the application of serotonin (red, *p* < 10^−6^, *n* = 47), but not for the saline (black, *p* = 0.70, *n* = 11). The change in mean firing with the iontophoretic application differs significantly between both conditions (*p* < 10^−4^, *n* = 47 for serotonin, *n* = 11 for saline, Wilcoxon rank-sum test). ***G***, The gain of the tuning curves decreases substantially more for the serotonin than for the saline condition (*p* = 0.006, *n* = 40 for serotonin, *n* = 5 for saline) while the subtractive change does not differ across conditions (*p* = 0.99, both Wilcoxon rank-sum test). ***H***, The response latency increases slightly for serotonin (*p* < 10^−3^, *n* = 45) but not for the saline condition (*p* = 0.55, *n* = 8). Note that due to the small sample for saline, this difference in modulation across conditions does not reach significance (*p* = 0.38). ***I***, The serotonin-induced changes in latency and mean firing rate are correlated (*r* = −0.51, *p* < 10^−3^, *n* = 45, Spearman's rank correlation).

We next wondered whether serotonin influenced the dynamics of the visual response, since previous work reported serotonergic influences on the dynamics of subcortical auditory processing in bats (Hurley and Pollak, 2005). To address this question, we used orientation subspace reverse correlation (Ringach et al., 1997), an approach that allows for detailed quantification of the dynamics of the orientation-selective response. The stimulus consisted of a random sequence of gratings of the same spatial frequency but different spatial phases and orientations, updated every 10 ms (see Materials and Methods). Figure 5*A* shows the average SDFs for each orientation for one example neuron for the baseline (top) and serotonin (bottom) blocks. We extracted tuning curves (“orientation subspace maps”) from these SDFs (Nienborg and Cumming, 2009; Fig. 5*B*) and quantified the changes in tuning as additive and multiplicative changes using type-II regression. Similar to the results for drifting gratings, we found a significant decrease in the mean response (Fig. 5*F*; *p* < 10^−4^, *n*_5-HT_ = 47, *n*_NaCl_ = 11; monkey M: *p* = 0.08, *n*_5-HT_ = 34, *n*_NaCl_ = 3; monkey K: *p* < 10^−3^, *n*_5-HT_ = 13, *n*_NaCl_ = 8) that was largely explained by multiplicative changes in the tuning curves (Fig. 5*G*; relative gain: *p* = 0.007, *n*_5-HT_ = 40, *n*_NaCl_ = 5; monkey M: *p* = 0.06, *n*_5-HT_ = 31, *n*_NaCl_ = 3; monkey K: *p* = 0.15, *n*_5-HT_ = 9, *n*_NaCl_ = 2; additive chane: *p* = 0.99, *n*_5-HT_ = 40, *n*_NaCl_ = 5; monkey M: *p* = 0.86, *n*_5-HT_ = 31, *n*_NaCl_ = 3; monkey K: *p* = 0.44, *n*_5-HT_ = 9, *n*_NaCl_ = 2). To explore the dynamics of the orientation-selective response, we computed the SD across the SDFs over time (Fig. 5*C*). For the example cell, we find that the response latency for the orientation-selective component of the response is slightly longer after serotonin application (Fig. 5*A*,*C*). Importantly, our control analysis (Fig. 5*D*,*E*; see Materials and Methods) reveals that this effect cannot be explained by the decreased signal-to-noise ratio owing to the reduced spike rate in the serotonin condition. Indeed, across the population, we observed a small but consistent and statistically significant increase in response latency (Fig. 5*H*; *p* < 10^−3^, *n*_5-HT_ = 45; monkey M: *p* < 0.01, *n*_5-HT_ = 33; monkey K: *p* = 0.02, *n*_5-HT_ = 12), which was correlated with the size of the serotonergic modulation in firing rate (Fig. 5*I*; *r* = −0.51, *p* < 10^−3^, *n*_5-HT_ = 45; monkey M: *r* = −0.56, *p* < 10^−3^, *n*_5-HT_ = 33; monkey K: *r* = −0.20, *p* = 0.54, *n*_5-HT_ = 12).

### The serotonin-induced changes differ quantitatively from contrast gain, but are accounted for by a simple membrane potential-based model

**Figure 6.**
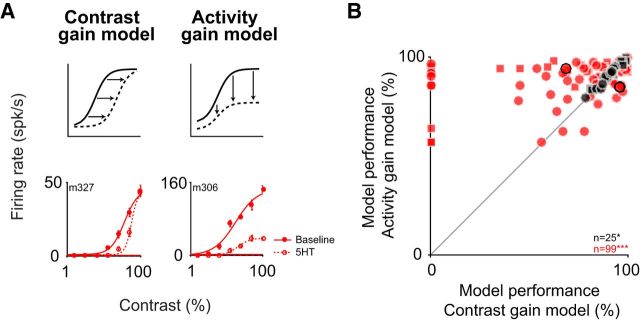
The serotonergic modulation cannot be accounted for by a contrast-gain model. ***A***, If the serotonergic modulation reflected a mechanism similar to contrast-gain control, the serotonergic modulation of the contrast tuning curve should result in a horizontal shift of the curve, without changing the response maximum (dashed line, top left), and an example neuron (bottom left) is compatible with such a change. Alternatively, under an activity-gain model (top right) serotonin application would result in a multiplicative downscaling of the tuning curve, as found for the example (bottom right). ***B***, Symbols are the same as in Figure 1. The activity-gain model in most cases explains a higher percentage of the variance than the contrast-gain model (*p* < 10^−6^, *n* = 99; Wilcoxon paired test). The change in variance explained differs significantly (*p* = 0.02, *n* = 99 for serotonin, *n* = 25 for saline, Wilcoxon rank-sum test) between the serotonin (5HT; red) and the saline control condition (NaCl; black). Example units in ***A*** are marked by the black circles.

The main serotonin-dependent changes of the tuning curves (i.e., a largely divisive change in the response and a slight increase in the response latency) are reminiscent of the divisive reduction and phase delay when lowering contrast (Carandini et al., 1997), which are accounted for by a model using divisive normalization (Heeger, 1992; Carandini et al., 1997). We therefore wondered whether the serotonin-induced changes mimic a reduction in contrast, suggesting it may engage a mechanism similar to contrast normalization. To test this hypothesis, we therefore compared the performance of two descriptive models, a contrast-gain model and an activity-gain model (Fig. 6*A*). For the contrast-gain model, the modulation by serotonin would only mimic a change in contrast, resulting in a horizontal shift of the tuning curve (Fig. 6*A*, left). Conversely, for the activity-gain model, the modulation by serotonin would result in a downscaling of the entire tuning curve (Fig. 6*A*, right). While the contrast-gain model provided a better fit to the data for a small number of cells in support of the hypothesis that the modulation by serotonin engages a similar mechanism as contrast, the activity-gain model performed substantially better in most cases (Fig. 6*B*; *p* < 10^−6^, *n*_5-HT_ = 99; monkey M: *p* < 0.01, *n*_5-HT_ = 66; monkey K: *p* < 10^−4^, *n*_5-HT_ = 33; Wilcoxon paired signed-rank test). This indicates that modulation by serotonin relies on a mechanism that differs from contrast-gain control.

**Figure 7.**
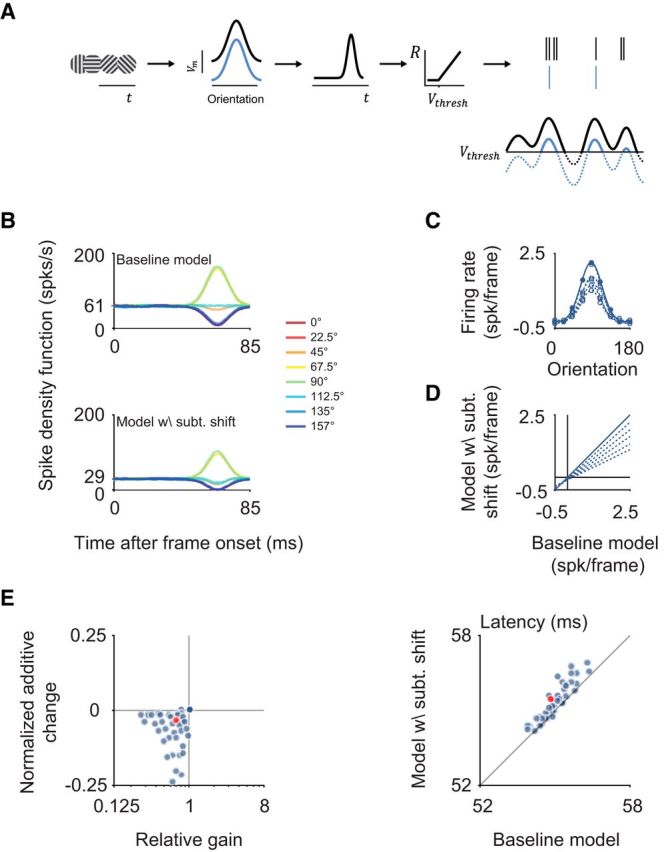
A simple threshold-linear model can account for the observed effects. ***A***, Schematic of the model: the orientation-selective response of the membrane potential to the stimulus sequence is temporally low-pass filtered before being passed through a threshold-linear nonlinearity and a Poisson process to generate spikes. The only effect by serotonin is a subtractive shift to the membrane potential (blue lines) that is equivalent to a higher value of the threshold (*V*_thresh_). ***B***, Example SDFs for the modeled baseline condition (top) and after a subtractive shift to the membrane potential aimed at mimicking the serotonin condition (bottom). ***C***, Tuning curves for the baseline condition (solid) and for subtractive shifts of different sizes (dotted) are shown. ***D***, Best fitting regression lines to quantify additive and subtractive changes are shown for different models. ***E***, The subtractive change in the model leads to gain changes, additive changes (left), as well as latency shifts (right) in the range (blue data points) observed in our data (red circles).

Finally we wondered whether a simple membrane potential-based model could account for the divisive change in the response and the slight increase in response latency. Specifically, we explored whether a linear change at the level of the membrane potential would suffice to account for the observed effects by serotonin (Fig. 7). This membrane potential-based model consisted of an orientation-selective response at the level of the membrane potential followed by a temporal low-pass filter to fit the orientation bandwidth and average latency of the neuronal response to the stimulus used for orientation subspace reverse correlation (see Materials and Methods; Fig. 7*A*). The time-varying response of the membrane potential was then passed through a threshold-linear spiking nonlinearity. To account for the effect of serotonin, a subtractive shift was applied to the membrane potential [Fig. 7*A*, *V_m_*(*t*)], moving it further away from the spiking threshold. Note that, while biophysically different, in our model this shift is equivalent to increasing the spiking threshold (*V_thresh_*) of the spiking nonlinearity. We found that this shift could account for the observed changes in gain, the additive changes (Fig. 7*D*), as well as the small increase in latency (Fig. 7*B*,*E*). A simple subtractive change of a threshold-linear spiking nonlinearity can therefore capture the serotonin-induced modulation of the visual responses.

### Serotonin has no systematic effect on response variability or covariability

**Figure 8.**
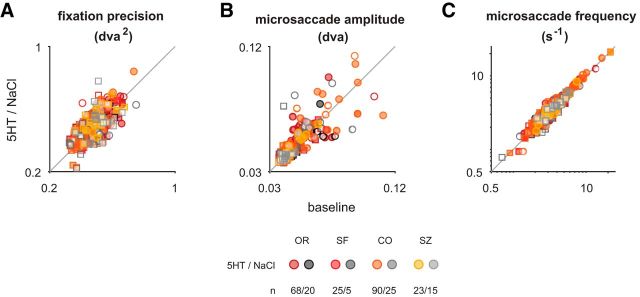
No systematic difference in eye movements between drug and baseline conditions. ***A***, Fixation precision (see Materials and Methods) did not differ between conditions [median 0.49 degrees of visual angle squared (dva^2^) for both baseline and 5-HT application, respectively; median 0.49 dva^2^ and 0.50 dva^2^ for baseline and saline application, respectively; the change with drug application did not differ significantly between the saline and 5-HT experiments, *p* = 0.68]. ***B***, Microsaccade amplitudes were similar between conditions (median 0.04 dva for baseline and serotonin application, respectively, *n*_5-HT_ = 206; median 0.04 dva for baseline and saline application, respectively, *n*_NaCl_ = 65) and their changes did not differ significantly for serotonin versus saline application (*p* = 0.43). **C**, Similarly, the frequency of microsaccades did not change between conditions (median, 3.4 s^−1^ for baseline and serotonin application, respectively, *n*_5-HT_ = 206; median, 3.2 and 3.3 s^−1^ for baseline and saline application, respectively), and the changes did not differ between conditions (*p* = 0.96; *n*_5-HT_ = 206, *n*_NaCl_ = 65, Wilcoxon rank-sum test for all comparisons). Note that for units for which several experiments were performed, only the first experiment was included. Symbols are the same as in Figure 1. Shading represents the type of experiment for which the modulation by drug application [serotonin (5HT) or saline (NaCl)] was quantified.

**Figure 9.**
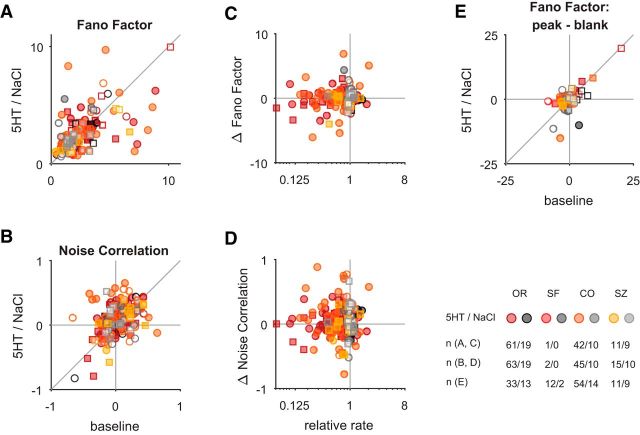
Serotonin application does not systematically change the Fano factor or noise correlation. Warm and gray colors depict experiments with serotonin (5HT) and saline (NaCl) application, respectively, as indicated. The number of units per experiment and condition included are specified. Filled symbols depict units with significant modulation (compare Fig. 1*E*). Circles and squares correspond to data from monkey M and K, respectively. ***A***, The variability of the neuronal response quantified by the Fano factor does not change systematically for the application of serotonin orientation: *p* = 0.77, *n* = 61 for serotonin; *p* = 0.55, *n* = 19 for saline; spatial frequency: *n* = 1 for serotonin; *n* = 0 for saline; contrast: *p* = 0.81, *n* = 42 for serotonin; *p* = 0.13, *n* = 10 for saline; size: *p* = 0.17, *n* = 11 for serotonin; *p* = 0.57, *n* = 9 for saline; all Wilcoxon paired signed-rank test). Note the similar distribution for serotonin and saline application in ***A–D***. ***B***, The single-unit multiunit noise correlation (corrected for changes in firing rate; see Materials and Methods) is compared for the baseline condition (abscissa) and the application of serotonin or saline (ordinate). The serotonin application did not result in a significant change of noise correlation across the population (orientation: *p* = 0.74, *n* = 63 for serotonin; *p* = 0.15, *n* = 19 for saline; spatial frequency: *n* = 2 for serotonin; *n* = 0 for saline; contrast: *p* = 0.07, *n* = 45 for serotonin; *p* = 0.38, *n* = 10 for saline; size: *p* = 0.59, *n* = 15 for serotonin; *p* = 0.43, *n* = 10 for saline; all Wilcoxon paired signed-rank test). The change in noise correlation (***D***) and Fano factor (***C***), respectively, are not systematically associated with the serotonergic modulation of a neuron's mean response (*p* > 0.20 for all Spearman's rank correlations for serotonin and *p* > 0.06 for all equivalent comparisons for saline application). ***E***, Serotonin also did not result in a significantly different change in the difference in Fano factor for the peak versus blank response compared with the saline application (*p* = 0.15, *n*_5-HT_ = 110, *n*_NaCl_ = 38). Note that for this analysis the preferred and blank stimuli had to be presented ≥4 times each for each unit.

Neuromodulators, such as acetylcholine, have been implicated in affecting not only the magnitude (Disney et al., 2007) but also the variability of sensory responses (Pinto et al., 2013), and it has been hypothesized that serotonin mediates an additional, acetylcholine-independent, mechanism of response desynchronization (Harris and Thiele, 2011). To test for this possibility, we measured the stimulus-independent response covariability [“noise-correlation” (Bair et al., 2001; Cohen and Kohn, 2011)] between the single-unit and the multiunit activity recorded on the same electrode. We note that measuring this correlation between single-unit and multiunit activity recorded on the same electrode has been shown to overestimate the absolute size of this correlation (Ecker et al., 2010). Nonetheless, this approach allows us to infer relative changes in noise correlation between the baseline and the serotonin condition. To reduce the effect of nonstationarities resulting from the onset of the serotonin application on noise correlations, we excluded the first 20 stimulus presentations of each experimental block for this analysis. Since fixational eye movements can affect the variability and covariability of visual neurons, we explored whether these differed systematically between the serotonin application and the saline controls. To this end, we compared the fixation precision (Cherici et al., 2012) as well as the frequency and amplitude of microsaccades within the fixation window (Fig. 8). For none of these metrics differed the modulation systematically between the serotonin and the saline application. Finally, we corrected noise correlation by that predicted from the change in firing rate (see Materials and Methods). In contrast to the hypothesis, we found no significant change in noise correlations for the serotonin condition (Fig. 9*B*; for orientation: *p* = 0.74, *n*_5-HT_ = 63 for serotonin; for spatial frequency: *n*_5-HT_ = 2 for serotonin; for contrast: *p* = 0.07, *n*_5-HT_ = 45 for serotonin; for size: *p* = 0.59, *n*_5-HT_ = 15 for serotonin; all Wilcoxon paired signed-rank test). We also did not observe a systematic relationship between the size of effect on firing rate and noise correlation (Fig. 9*D*; for orientation: *r* = 0.07, *p* = 0.57, *n*_5-HT_ = 63; for spatial frequency: *n*_5-HT_ = 2; for contrast: *r* = −0.02, *p* = 0.88, *n*_5-HT_ = 45; for size: *r* = −0.13, *p* = 0.66, *n*_5-HT_ = 15; all Spearman's rank correlation). Finally, we also explored the effect of serotonin on the variability of the sensory response, quantified as Fano factor, and observed no systematic change in the two conditions (Fig. 9*A*; for orientation: *p* = 0.77, *n*_5-HT_ = 61; for spatial frequency: *n*_5-HT_ = 1; for contrast: *p* = 0.81, *n*_5-HT_ = 42; for size: *p* = 0.17, *n*_5-HT_ = 11; all Wilcoxon paired signed-rank tests). Moreover, the change in mean firing rate was not associated with a systematic change in Fano factor (Fig. 9*C*; for orientation: *r* = −0.22, *p* = 0.08, *n*_5-HT_ = 61; for spatial frequency: n_5-HT_ = 1; for contrast: *r* = −0.14, *p* = 0.38, *n*_5-HT_ = 42; for size: *r* = 0.19, *p* = 0. 58, *n*_5-HT_ = 11; all Spearman's rank correlation). Additionally, there was no systematic change in the difference in Fano factor in response to a neuron's preferred stimulus compared with that to a blank stimulus (Fig. 9*E*). Together, these analyses indicate that the application of serotonin leaves the response variability and covariability in macaque V1 largely unchanged.

## Discussion

Here we combined extracellular recordings and iontophoresis in V1 of awake macaques to explore the modulatory effects of serotonin on visual processing. We found that across a variety of visual stimulus dimensions, the modulation by serotonin across the neuronal population was surprisingly uniform and (1) was dominated by a decrease in the neurons' response gain, (2) showed a slight slowing of the dynamics of the response, and (3) resulted in no systematic change of the neuronal variability, covariability, or stimulus selectivity. Our observed effects could be captured by a descriptive model in which serotonin caused a simple additive change at the level of the threshold-linear spiking nonlinearity.

### A surprisingly uniform effect despite receptor and cellular diversity

In this study, we focused on the serotonergic modulation in the awake macaque of functional tuning properties along four well characterized visual dimensions (orientation, spatial frequency, contrast, and size) that are encoded by neurons in V1. We focused on awake animals using the endogenous ligand while previous work examined the modulation to receptor-specific ligands in the striate cortex of anesthetized macaques and, in contrast with the predominantly suppressive effect we found, observed variable modulation (Watakabe et al., 2009). This apparent discrepancy may in part reflect a net effect between facilitation and suppression mediated by different receptor classes all activated by serotonin. Additionally, our recordings in the awake animal likely mimimized fluctuations in brain state found under anesthesia (Ecker et al., 2014) and avoided effects of anesthesia on serotonergic neurons (Johansen et al., 2015). Moreover, the focus on parametrized functional properties may have contributed to our ability to identify an effect—a gain decrease—that is surprisingly functionally uniform across the population and stimulus dimensions. This effect dominated despite variability on a neuron-by-neuron basis, which was also observed previously in anesthetized animals (Waterhouse et al., 1990; Watakabe et al., 2009). In light of the known diversity of receptor expression on different cell types in the macaque V1 (Watakabe et al., 2009), this main observed effect is likely mediated by different cellular or network mechanisms. For example, in the input layers of V1, receptors 5-HT_1B_ and 5-HT_2A_ are expressed by the majority of excitatory neurons, but not by GABAergic neurons, and are typically coexpressed by the same neurons in layer IVC (Watakabe et al., 2009). The predominantly divisive effect of serotonin may therefore result from an interaction of the receptor activation in excitatory neurons. A previous study applied a 5-HT_1A_ agonist to layers IV and V in V1 of anesthetized macaques and also observed a decrease of multiunit activity (Rauch et al., 2008). Given the lack of 5-HT_1A_-receptor expression in these layers in macaque V1 (Watakabe et al., 2009), this decrease in activity may at least partially reflect a cross-activation of the densely expressed 5-HT_1B_ receptors. This modulation was thought to be mediated by a hyperpolarization of the membrane potential, analogous to the membrane potential-based model we present here. Serotonin-mediated hyperpolarization of the membrane potential was also recently identified at the axon initial segment in auditory neurons in gerbils (Ko et al., 2016). Conversely, in layer II, where the receptors are expressed on a subset of both excitatory and inhibitory neurons, but typically not coexpressed (Watakabe et al., 2009), the decrease in gain may reflect network interactions between inhibition and excitation. Different circuit elements may also include vasoactive intestinal peptide-positive (VIP+) interneurons that express the 5-HT_3A_ receptor (Rudy et al., 2011), although in the macaque V1 5-HT_3A_-receptor expression has only been observed for layers 5/6 (Watakabe et al., 2009), while VIP+ interneurons are most pronounced in layers 2/3 (Gabbott and Bacon, 1997). The same functional computation—gain control—may therefore be implemented by different components of the neuronal hardware within the same neuromodulator system serotonin, at least on the time scale we examined.

Indeed, since we applied serotonin in blocks in the minute time scale, the modulation likely mimics changes in the tonic level of discharge of serotonergic neurons, implicated in signaling the contextual valence on such slow time scales, and providing different signals from the phasic responses (Cohen et al., 2015). Interestingly, for short (<1 s) phasic serotonergic stimulation, a divisive modulation was previously observed only for the spontaneous response, not the sensory-driven response, in the olfactory bulb in anesthetized mice (Lottem et al., 2016), in contrast with our findings. Conversely, a decrease in gain of the sensory response in the mouse olfactory bulb was observed for sustained (>30 s) serotonergic activation (Petzold et al., 2009), similar to the modulation observed in this study.

Behaviorally, decreasing the gain of a sensory response can contribute to a delay or reduction in the response to sensory stimulation, as has been previously observed for a reduced startle response in rats (Davis et al., 1980; Sipes and Geyer, 1994), or reduced mechanosensory responsivity in mice (Dugué et al., 2014). Although these behavioral findings have typically been interpreted in the context of a serotonergic role for motor or emotional processing (Crockett et al., 2009; Cools et al., 2011; Correia et al., 2017), the observed decreased sensory gain suggests at least partially a sensory involvement. It may also reflect a sensory signature of how serotonin promotes waiting (Miyazaki et al., 2014; Ranade et al., 2014; Fonseca et al., 2015) by lowering the salience of sensory input.

On this slow (minute) time scale, serotonin may be complementary to the action of cholinergic neuromodulation, which has been found to increase the gain of the visual input in macaque V1 (Disney et al., 2007), and has been linked to mediating spatial attention (Herrero et al., 2008). Notably, acetylcholine in macaque V1 was found to substantially modulate the gain but not the variability of the response (Herrero et al., 2008), a dissociation that is mirrored by our results here. It suggests that distinct mechanisms are available to modulate the variability and level of the sensory responses, although cognitive states, such as spatial attention, typically affect both (Cohen and Maunsell, 2009; Mitchell et al., 2009).

### Potential implications for the serotonergic role in visual hallucinations

An influential perspective of perception is that it reflects an inference process (Helmholtz, 1867; Gregory, 1980; Lee and Mumford, 2003; Yuille and Kersten, 2006), in which internal beliefs about the world are combined with the incoming sensory evidence (Lee and Mumford, 2003; Yuille and Kersten, 2006). Mounting physiological evidence suggests that some of this combination of internal (“top-down”) and external sensory signals occur already at the level of sensory neurons (Fiser et al., 2010; Nienborg and Roelfsema, 2015; Cumming and Nienborg, 2016). In this framework, psychiatric diseases such as schizophrenia (Friston, 2005) are associated with an imbalance between internally generated (top-down) and externally driven feed-forward signals. Hallucinations, for example, have been suggested to arise from an imbalance toward internally generated over externally driven sensory signals (Friston, 2005; Notredame et al., 2014; Jardri et al., 2016; Schmack et al., 2017). Such an imbalance may also explain, for example, visual hallucinations during visual impairment (Charles Bonnet syndrome; Gold and Rabins, 1989), when the feed-forward visual input is degraded. Similarly, decreasing the gain of the visual input could shift the balance toward the internally generated signals, and thus result in visual hallucinations. The decrease in gain by serotonin we found here may therefore shed light onto the mechanism by which the visual cortex is involved in hallucinations caused by serotonergic hallucinogens (Bressloff et al., 2002; de Araujo et al., 2012; Kometer et al., 2013; Carhart-Harris et al., 2016; Kometer and Vollenweider, 2016).

### Conclusion

To our knowledge this is the first study to explore the role of serotonergic modulation of neuronal activity in the sensory cortex of awake animals. The modulatory effect we observed across the population was surprisingly homogeneous—a simple decrease in the response gain of the neural activity. Such gain modulation is an important component of the cortical computation (Salinas and Thier, 2000) that controls the responses without changing the receptive field properties. It is therefore well suited to modulate the responses according to the animal's internal state, e.g. influenced by the valence of the contextual environment (Cohen et al., 2015).

## References

[B1] Albrecht DG, Hamilton DB (1982) Striate cortex of monkey and cat: contrast response function. J Neurophysiol 48:217–237. 7119846 10.1152/jn.1982.48.1.217

[B2] Atallah BV, Bruns W, Carandini M, Scanziani M (2012) Parvalbumin-expressing interneurons linearly transform cortical responses to visual stimuli. Neuron 73:159–170. 10.1016/j.neuron.2011.12.013 22243754 PMC3743079

[B3] Bachatene L, Bharmauria V, Cattan S, Molotchnikoff S (2013) Fluoxetine and serotonin facilitate attractive-adaptation-induced orientation plasticity in adult cat visual cortex. Eur J Neurosci 38:2065–2077. 10.1111/ejn.12206 23581614

[B4] Bair W, Zohary E, Newsome WT (2001) Correlated firing in macaque visual area MT: time scales and relationship to behavior. J Neurosci 21:1676–1697. 11222658 10.1523/JNEUROSCI.21-05-01676.2001 PMC6762960

[B5] Bracewell RN (1986) The Fourier transform and its applications. Singapore: McGraw-Hill.

[B6] Brainard DH (1997) The psychophysics toolbox. Spat Vis 10:433–436. 9176952

[B7] Bressloff PC, Cowan JD, Golubitsky M, Thomas PJ, Wiener MC (2002) What geometric visual hallucinations tell us about the visual cortex. Neural Comput 14:473–491. 11860679 10.1162/089976602317250861

[B8] Carandini M (2004) Amplification of trial-to-trial response variability by neurons in visual cortex. PLoS Biol 2:E264. 10.1371/journal.pbio.0020264 15328535 PMC509408

[B9] Carandini M, Ferster D (2000) Membrane potential and firing rate in cat primary visual cortex. J Neurosci 20:470–484. 10627623 10.1523/JNEUROSCI.20-01-00470.2000 PMC6774139

[B10] Carandini M, Heeger DJ, Movshon JA (1997) Linearity and normalization in simple cells of the macaque primary visual cortex. J Neurosci 17:8621–8644. 9334433 10.1523/JNEUROSCI.17-21-08621.1997 PMC6573724

[B11] Carhart-Harris RL, Muthukumaraswamy S, Roseman L, Kaelen M, Droog W, Murphy K, Tagliazucchi E, Schenberg EE, Nest T, Orban C, Leech R, Williams LT, Williams TM, Bolstridge M, Sessa B, McGonigle J, Sereno MI, Nichols D, Hellyer PJ, Hobden P, et al (2016) Neural correlates of the LSD experience revealed by multimodal neuroimaging. Proc Natl Acad Sci U S A 113:4853–4858. 10.1073/pnas.1518377113 27071089 PMC4855588

[B12] Cavanaugh JR, Bair W, Movshon JA (2002) Nature and interaction of signals from the receptive field center and surround in macaque V1 neurons. J Neurophysiol 88:2530–2546. 10.1152/jn.00692.2001 12424292

[B13] Cherici C, Kuang X, Poletti M, Rucci M (2012) Precision of sustained fixation in trained and untrained observers. J Vis 12(6):pii:31. 10.1167/12.6.31 22728680 PMC3489479

[B14] Clery S, Cumming BG, Nienborg H (2017) Decision-related activity in macaque V2 for fine disparity discrimination is not compatible with optimal linear read-out. J Neurosci 37:715–725. 10.1523/JNEUROSCI.2445-16.2016 28100751 PMC5242413

[B15] Cohen JY, Amoroso MW, Uchida N (2015) Serotonergic neurons signal reward and punishment on multiple timescales. Elife 4. 10.7554/eLife.06346 25714923 PMC4389268

[B16] Cohen MR, Kohn A (2011) Measuring and interpreting neuronal correlations. Nat Neurosci 14:811–819. 10.1038/nn.2842 21709677 PMC3586814

[B17] Cohen MR, Maunsell JH (2009) Attention improves performance primarily by reducing interneuronal correlations. Nat Neurosci 12:1594–1600. 10.1038/nn.2439 19915566 PMC2820564

[B18] Cools R, Nakamura K, Daw ND (2011) Serotonin and dopamine: unifying affective, activational, and decision functions. Neuropsychopharmacology 36:98–113. 10.1038/npp.2010.121 20736991 PMC3055512

[B19] Correia PA, Lottem E, Banerjee D, Machado AS, Carey MR, Mainen ZF (2017) Transient inhibition and long-term facilitation of locomotion by phasic optogenetic activation of serotonin neurons. Elife 6:pii:e20975. 10.7554/eLife.20975 28193320 PMC5308893

[B20] Crockett MJ, Clark L, Robbins TW (2009) Reconciling the role of serotonin in behavioral inhibition and aversion: acute tryptophan depletion abolishes punishment-induced inhibition in humans. J Neurosci 29:11993–11999. 10.1523/JNEUROSCI.2513-09.2009 19776285 PMC2775933

[B21] Cumming BG, Nienborg H (2016) Feedforward and feedback sources of choice probability in neural population responses. Curr Opin Neurobiol 37:126–132. 10.1016/j.conb.2016.01.009 26922005 PMC4927695

[B22] Davis M, Strachan DI, Kass E (1980) Excitatory and inhibitory effects of serotonin on sensorimotor reactivity measured with acoustic startle. Science 209:521–523. 10.1126/science.7394520 7394520

[B23] Dayan P (2012) Twenty-five lessons from computational neuromodulation. Neuron 76:240–256. 10.1016/j.neuron.2012.09.027 23040818

[B24] Dayan P, Huys Q (2015) Serotonin's many meanings elude simple theories. Elife 4. 10.7554/eLife.07390 25853523 PMC4389267

[B25] de Araujo DB, Ribeiro S, Cecchi GA, Carvalho FM, Sanchez TA, Pinto JP, de Martinis BS, Crippa JA, Hallak JE, Santos AC (2012) Seeing with the eyes shut: neural basis of enhanced imagery following Ayahuasca ingestion. Hum Brain Mapp 33: 2550–2560. 10.1002/hbm.21381 21922603 PMC6870240

[B26] de Lima AD, Bloom FE, Morrison JH (1988) Synaptic organization of serotonin-immunoreactive fibers in primary visual cortex of the macaque monkey. J Comp Neurol 274:280–294. 10.1002/cne.902740211 3209742

[B27] Disney AA, Aoki C, Hawken MJ (2007) Gain modulation by nicotine in macaque V1. Neuron 56:701–713. 10.1016/j.neuron.2007.09.034 18031686 PMC2875676

[B28] Dugué GP, Lörincz ML, Lottem E, Audero E, Matias S, Correia PA, Léna C, Mainen ZF (2014) Optogenetic recruitment of dorsal raphe serotonergic neurons acutely decreases mechanosensory responsivity in behaving mice. PLoS One 9:e105941. 10.1371/journal.pone.0105941 25148042 PMC4141837

[B29] Eastman KM, Huk AC (2012) PLDAPS: a hardware architecture and software toolbox for neurophysiology requiring complex visual stimuli and online behavioral control. Front Neuroinform 6:1. 10.3389/fninf.2012.00001 22319490 PMC3269100

[B30] Ecker AS, Berens P, Keliris GA, Bethge M, Logothetis NK, Tolias AS (2010) Decorrelated neuronal firing in cortical microcircuits. Science 327:584–587. 10.1126/science.1179867 20110506

[B31] Ecker AS, Berens P, Cotton RJ, Subramaniyan M, Denfield GH, Cadwell CR, Smirnakis SM, Bethge M, Tolias AS (2014) State dependence of noise correlations in macaque primary visual cortex. Neuron 82:235–248. 10.1016/j.neuron.2014.02.006 24698278 PMC3990250

[B32] Fiser J, Berkes P, Orbán G, Lengyel M (2010) Statistically optimal perception and learning: from behavior to neural representations. Trends Cogn Sci 14:119–130. 10.1016/j.tics.2010.01.003 20153683 PMC2939867

[B33] Fonseca MS, Murakami M, Mainen ZF (2015) Activation of dorsal raphe serotonergic neurons promotes waiting but is not reinforcing. Curr Biol 25:306–315. 10.1016/j.cub.2014.12.002 25601545

[B34] Friedman HS, Priebe CE (1998) Estimating stimulus response latency. J Neurosci Methods 83:185–194. 10.1016/S0165-0270(98)00075-2 9765132

[B35] Friston K (2005) Disconnection and cognitive dysmetria in schizophrenia. Am J Psychiatry 162:429–432. 10.1176/appi.ajp.162.3.429 15741456

[B36] Gabbott PL, Bacon SJ (1997) Vasoactive intestinal polypeptide containing neurones in monkey medial prefrontal cortex (mPFC): colocalisation with calretinin. Brain Res 744:179–184. 10.1016/S0006-8993(96)01232-2 9030431

[B37] Gold K, Rabins PV (1989) Isolated visual hallucinations and the Charles Bonnet syndrome: a review of the literature and presentation of six cases. Compr Psychiatry 30:90–98. 10.1016/0010-440X(89)90122-3 2647403

[B38] Goris RL, Movshon JA, Simoncelli EP (2014) Partitioning neuronal variability. Nat Neurosci 17:858–865. 10.1038/nn.3711 24777419 PMC4135707

[B39] Gregory RL (1980) Perceptions as hypotheses. Philos Trans R Soc Lond B Biol Sci 290:181–197. 10.1098/rstb.1980.0090 6106237

[B40] Gur M, Kagan I, Snodderly DM (2005) Orientation and direction selectivity of neurons in V1 of alert monkeys: functional relationships and laminar distributions. Cereb Cortex 15:1207–1221. 10.1093/cercor/bhi003 15616136

[B41] Harris KD, Thiele A (2011) Cortical state and attention. Nat Rev Neurosci 12:509–523. 10.1038/nrn3084 21829219 PMC3324821

[B42] Hayashi K, Nakao K, Nakamura K (2015) Appetitive and aversive information coding in the primate dorsal raphé nucleus. J Neurosci 35:6195–6208. 10.1523/JNEUROSCI.2860-14.2015 25878290 PMC6605165

[B43] Heeger DJ (1992) Normalization of cell responses in cat striate cortex. Vis Neurosci 9:181–197. 10.1017/S0952523800009640 1504027

[B44] Helmholtz H (1867) Handbuch der physiologischen optik. Leipzig: Voss.

[B45] Herrero JL, Roberts MJ, Delicato LS, Gieselmann MA, Dayan P, Thiele A (2008) Acetylcholine contributes through muscarinic receptors to attentional modulation in V1. Nature 454:1110–1114. 10.1038/nature07141 18633352 PMC2666819

[B46] Hurley LM, Pollak GD (2005) Serotonin shifts first-spike latencies of inferior colliculus neurons. J Neurosci 25:7876–7886. 10.1523/JNEUROSCI.1178-05.2005 16120790 PMC6725259

[B47] Hurley LM, Devilbiss DM, Waterhouse BD (2004) A matter of focus: monoaminergic modulation of stimulus coding in mammalian sensory networks. Curr Opin Neurobiol 14:488–495. 10.1016/j.conb.2004.06.007 15321070

[B48] Jacob SN, Ott T, Nieder A (2013) Dopamine regulates two classes of primate prefrontal neurons that represent sensory signals. J Neurosci 33:13724–13734. 10.1523/JNEUROSCI.0210-13.2013 23966694 PMC6618653

[B49] Jacobs BL, Azmitia EC (1992) Structure and function of the brain serotonin system. Physiol Rev 72:165–229. 1731370 10.1152/physrev.1992.72.1.165

[B50] Jardri R, Hugdahl K, Hughes M, Brunelin J, Waters F, Alderson-Day B, Smailes D, Sterzer P, Corlett PR, Leptourgos P, Debbané M, Cachia A, Denève S (2016) Are hallucinations due to an imbalance between excitatory and inhibitory influences on the brain. Schizophr Bull 42:1124–1134. 10.1093/schbul/sbw075 27261492 PMC4988749

[B51] Johansen SL, Iceman KE, Iceman CR, Taylor BE, Harris MB (2015) Isoflurane causes concentration-dependent inhibition of medullary raphé 5-HT neurons *in situ*. Auton Neurosci 193:51–56. 10.1016/j.autneu.2015.07.002 26213357 PMC4658272

[B52] Kleiner M, Brainard DH, Pelli DG (2007) What's new in Psychtoolbox-3? Perception 36, ECVP Abstract Supplement.

[B53] Ko KW, Rasband MN, Meseguer V, Kramer RH, Golding NL (2016) Serotonin modulates spike probability in the axon initial segment through HCN channels. Nat Neurosci 19:826–834. 10.1038/nn.4293 27110919 PMC4882252

[B54] Kometer M, Vollenweider FX (2016) Serotonergic hallucinogen-induced visual perceptual alterations. Curr Top Behav Neurosci. Advance online publication. Retrieved October 19, 2017. 10.1007/7854.2016.461 27900674

[B55] Kometer M, Schmidt A, Jäncke L, Vollenweider FX (2013) Activation of serotonin 2A receptors underlies the psilocybin-induced effects on α oscillations, N170 visual-evoked potentials, and visual hallucinations. J Neurosci 33:10544–10551. 10.1523/JNEUROSCI.3007-12.2013 23785166 PMC6618596

[B56] Lee J, Williford T, Maunsell JH (2007) Spatial attention and the latency of neuronal responses in macaque area V4. J Neurosci 27:9632–9637. 10.1523/JNEUROSCI.2734-07.2007 17804623 PMC6672969

[B57] Lee TS, Mumford D (2003) Hierarchical Bayesian inference in the visual cortex. J Opt Soc Am A Opt Image Sci Vis 20:1434–1448. 10.1364/JOSAA.20.001434 12868647

[B58] Lottem E, Lörincz ML, Mainen ZF (2016) Optogenetic activation of dorsal raphe serotonin neurons rapidly inhibits spontaneous but not odor-evoked activity in olfactory cortex. J Neurosci 36:7–18. 10.1523/JNEUROSCI.3008-15.2016 26740645 PMC6601795

[B59] Maya Vetencourt JF, Sale A, Viegi A, Baroncelli L, De Pasquale R, O'Leary OF, Castrén E, Maffei L (2008) The antidepressant fluoxetine restores plasticity in the adult visual cortex. Science 320:385–388. 10.1126/science.1150516 18420937

[B60] McAdams CJ, Maunsell JH (1999) Effects of attention on orientation-tuning functions of single neurons in macaque cortical area V4. J Neurosci 19:431–441. 9870971 10.1523/JNEUROSCI.19-01-00431.1999 PMC6782389

[B61] Mitchell JF, Sundberg KA, Reynolds JH (2009) Spatial attention decorrelates intrinsic activity fluctuations in macaque area V4. Neuron 63:879–888. 10.1016/j.neuron.2009.09.013 19778515 PMC2765230

[B62] Miyazaki KW, Miyazaki K, Tanaka KF, Yamanaka A, Takahashi A, Tabuchi S, Doya K (2014) Optogenetic activation of dorsal raphe serotonin neurons enhances patience for future rewards. Curr Biol 24:2033–2040. 10.1016/j.cub.2014.07.041 25155504

[B63] Nienborg H, Cumming BG (2006) Macaque V2 neurons, but not V1 neurons, show choice-related activity. J Neurosci 26:9567–9578. 10.1523/JNEUROSCI.2256-06.2006 16971541 PMC6674586

[B64] Nienborg H, Cumming BG (2009) Decision-related activity in sensory neurons reflects more than a neuron's causal effect. Nature 459:89–92. 10.1038/nature07821 19270683 PMC2917918

[B65] Nienborg H, Cumming BG (2014) Decision-related activity in sensory neurons may depend on the columnar architecture of cerebral cortex. J Neurosci 34:3579–3585. 10.1523/JNEUROSCI.2340-13.2014 24599457 PMC3942575

[B66] Nienborg H, Roelfsema PR (2015) Belief states as a framework to explain extra-retinal influences in visual cortex. Curr Opin Neurobiol 32:45–52. 10.1016/j.conb.2014.10.013 25463564

[B67] Nienborg H, Bridge H, Parker AJ, Cumming BG (2004) Receptive field size in V1 neurons limits acuity for perceiving disparity modulation. J Neurosci 24:2065–2076. 10.1523/JNEUROSCI.3887-03.2004 14999058 PMC6730443

[B68] Nienborg H, Hasenstaub A, Nauhaus I, Taniguchi H, Huang ZJ, Callaway EM (2013) Contrast dependence and differential contributions from somatostatin- and parvalbumin-expressing neurons to spatial integration in mouse V1. J Neurosci 33:11145–11154. 10.1523/JNEUROSCI.5320-12.2013 23825418 PMC3718383

[B69] Notredame CE, Pins D, Deneve S, Jardri R (2014) What visual illusions teach us about schizophrenia. Front Integr Neurosci 8:63. 10.3389/fnint.2014.00063 25161614 PMC4130106

[B70] Ott T, Jacob SN, Nieder A (2014) Dopamine receptors differentially enhance rule coding in primate prefrontal cortex neurons. Neuron 84:1317–1328. 10.1016/j.neuron.2014.11.012 25482027

[B71] Pelli DG (1997) The VideoToolbox software for visual psychophysics: transforming numbers into movies. Spat Vis 10:437–442. 10.1163/156856897X00366 9176953

[B72] Petzold GC, Hagiwara A, Murthy VN (2009) Serotonergic modulation of odor input to the mammalian olfactory bulb. Nat Neurosci 12:784–791. 10.1038/nn.2335 19430472

[B73] Pinto L, Goard MJ, Estandian D, Xu M, Kwan AC, Lee SH, Harrison TC, Feng G, Dan Y (2013) Fast modulation of visual perception by basal forebrain cholinergic neurons. Nat Neurosci 16:1857–1863. 10.1038/nn.3552 24162654 PMC4201942

[B74] Priebe NJ, Ferster D (2008) Inhibition, spike threshold, and stimulus selectivity in primary visual cortex. Neuron 57:482–497. 10.1016/j.neuron.2008.02.005 18304479

[B75] Rabinowitz NC, Goris RL, Cohen M, Simoncelli E (2015) Attention stabilizes the shared gain of V4 populations. Elife 4:e08998. 10.7554/eLife.08998 26523390 PMC4758958

[B76] Ranade S, Pi HJ, Kepecs A (2014) Neuroscience: waiting for serotonin. Curr Biol 24:R803–R805. 10.1016/j.cub.2014.07.024 25202872

[B77] Ranade SP, Mainen ZF (2009) Transient firing of dorsal raphe neurons encodes diverse and specific sensory, motor, and reward events. J Neurophysiol 102:3026–3037. 10.1152/jn.00507.2009 19710375

[B78] Rauch A, Rainer G, Logothetis NK (2008) The effect of a serotonin-induced dissociation between spiking and perisynaptic activity on BOLD functional MRI. Proc Natl Acad Sci U S A 105:6759–6764. 10.1073/pnas.0800312105 18456837 PMC2373337

[B79] Ringach DL, Hawken MJ, Shapley R (1997) Dynamics of orientation tuning in macaque primary visual cortex. Nature 387:281–284. 10.1038/387281a0 9153392

[B80] Ringach DL, Shapley RM, Hawken MJ (2002) Orientation selectivity in macaque V1: diversity and laminar dependence. J Neurosci 22:5639–5651. 12097515 10.1523/JNEUROSCI.22-13-05639.2002 PMC6758222

[B81] Rudy B, Fishell G, Lee S, Hjerling-Leffler J (2011) Three groups of interneurons account for nearly 100% of neocortical GABAergic neurons. Dev Neurobiol 71:45–61. 10.1002/dneu.20853 21154909 PMC3556905

[B82] Salinas E, Thier P (2000) Gain modulation: a major computational principle of the central nervous system. Neuron 27:15–21. 10.1016/S0896-6273(00)00004-0 10939327

[B83] Schmack K, Rothkirch M, Priller J, Sterzer P (2017) Enhanced predictive signalling in schizophrenia. Hum Brain Mapp 38:1767–1779. 10.1002/hbm.23480 28097738 PMC6866905

[B84] Schmitzer-Torbert N, Jackson J, Henze D, Harris K, Redish AD (2005) Quantitative measures of cluster quality for use in extracellular recordings. Neuroscience 131:1–11. 10.1016/j.neuroscience.2004.09.066 15680687

[B85] Sipes TA, Geyer MA (1994) Multiple serotonin receptor subtypes modulate prepulse inhibition of the startle response in rats. Neuropharmacology 33:441–448. 10.1016/0028-3908(94)90074-4 7984282

[B86] Skottun BC, De Valois RL, Grosof DH, Movshon JA, Albrecht DG, Bonds AB (1991) Classifying simple and complex cells on the basis of response modulation. Vision Res 31:1079–1086. 1909826 10.1016/0042-6989(91)90033-2

[B87] Thiele A, Delicato LS, Roberts MJ, Gieselmann MA (2006) A novel electrode-pipette design for simultaneous recording of extracellular spikes and iontophoretic drug application in awake behaving monkeys. J Neurosci Methods 158:207–211. 10.1016/j.jneumeth.2006.05.032 16843532 PMC2666830

[B88] Watakabe A, Komatsu Y, Sadakane O, Shimegi S, Takahata T, Higo N, Tochitani S, Hashikawa T, Naito T, Osaki H, Sakamoto H, Okamoto M, Ishikawa A, Hara S, Akasaki T, Sato H, Yamamori T (2009) Enriched expression of serotonin 1B and 2A receptor genes in macaque visual cortex and their bidirectional modulatory effects on neuronal responses. Cereb Cortex 19:1915–1928. 10.1093/cercor/bhn219 19056862 PMC2705701

[B89] Waterhouse BD, Azizi SA, Burne RA, Woodward DJ (1990) Modulation of rat cortical area 17 neuronal responses to moving visual stimuli during norepinephrine and serotonin microiontophoresis. Brain Res 514:276–292. 10.1016/0006-8993(90)91422-D 2357542

[B90] Williams GV, Rao SG, Goldman-Rakic PS (2002) The physiological role of 5-HT_2A_ receptors in working memory. J Neurosci 22:2843–2854. 11923449 10.1523/JNEUROSCI.22-07-02843.2002 PMC6758292

[B91] Williford T, Maunsell JH (2006) Effects of spatial attention on contrast response functions in macaque area V4. J Neurophysiol 96:40–54. 10.1152/jn.01207.2005 16772516

[B92] Yuille A, Kersten D (2006) Vision as Bayesian inference: analysis by synthesis? Trends Cogn Sci 10:301–308. 10.1016/j.tics.2006.05.002 16784882

